# Validation of malaria-attributed deaths using verbal autopsy studies: a systematic review

**DOI:** 10.1186/s12936-024-05035-5

**Published:** 2024-07-19

**Authors:** Ronald Carshon-Marsh, Susan Bondy, Theodore Witek, Prabhat Jha

**Affiliations:** 1 Dalla Lana School of Public Health, University of Toronto, Toronto, ON M5T 3M7 Canada; 2 Centre for Global Health Research, Unity Health Toronto, Toronto, ON M5B 1W8 Canada

**Keywords:** Verbal autopsy, Minimally invasive tissue sampling, Complete diagnostic autopsy, CHAMPS, Malaria, Cause of death, Sub-Saharan Africa, Clinical malaria, Malaria infection

## Abstract

**Background:**

Malaria contributes substantially to the persistent burden of child deaths in sub-Saharan Africa. Accurate and comprehensive malaria mortality data are crucial to monitor the progress in reducing malaria incidence and mortality. Verbal Autopsy (VA) ascertains the cause of death despite its limitations leading to misclassification errors. Minimally Invasive Tissue Sampling (MITS) is being conducted in some settings as an alternative to Complete Diagnostic Autopsy (CDA). The present study examines the validity of malaria-related deaths comparing VA diagnoses with those obtained through MITS and/or CDA.

**Methods:**

A comprehensive literature search for original studies in English language using Ovid MEDLINE, Ovid Embase, CINAHL via EBSCO, Scopus, The Cochrane Library via Wiley, Google Scholar and searching the MITS Surveillance Alliance papers was carried out. The reference period was January 1, 1990–March 31, 2024. The Preferred Reporting Items for Systematic Reviews and Meta-Analyses guidelines were adopted.

**Results:**

Among 71 articles identified in the databases, 21 matched the eligibility criteria. Qualitative syntheses showed that malaria Cause Specific Mortality Fractions (CSMFs) across various studies ranged from 2 to 31%. *Plasmodium falciparum* was mostly responsible for these deaths and the most common complications were anaemia and cerebral malaria. The sensitivity and specificity of the VA validation studies ranged from 18.4% to 33% and from 86.6% to 97%, respectively, and there was a high level of misclassification for both InSilico and Expert Algorithm VA for malaria compared to MITS. The overall concordance rates between MITS and CDA diagnoses ranged from 68 to 90%, with the highest concordance seen in deaths due to infectious diseases and malignant tumours. Clinical data increased diagnostic coincidence between MITS blind to clinical data and the gold standard CDA by 11%.

**Conclusions:**

The comprehensive review finds that MITS demonstrated better accuracy compared to VA in diagnosing malaria-attributed deaths, particularly in hospital settings. The high specificity of malaria in VA diagnosis suggests population-based estimates of the proportion of deaths due to malaria are broadly plausible.

**Supplementary Information:**

The online version contains supplementary material available at 10.1186/s12936-024-05035-5.

## Background

Child mortality is decreasing globally, but nonetheless an estimated 5 million children under-five years of age died from various causes in 2021 [1]. A significant portion of the 3 million child deaths occurring in sub-Saharan Africa are attributable to the persistent burden of malaria. In 2021, the global toll of malaria-related deaths was estimated at 619,000 [2], with the overwhelming majority occurring in African children [2].

Accurate and comprehensive malaria mortality data are crucial at both national and subnational levels to monitor the progress in reducing malaria incidence and mortality and to enable effective policymaking and programme planning [3]. However, civil registration and vital statistics are weak and incomplete in many low- and middle-income countries (LMICs), especially in sub-Saharan Africa. The majority of deaths in these regions occur outside of healthcare facilities, predominantly at home, where access to medical care and documentation of causes of death are limited. Consequently, there is a dearth of medically-certified information on malaria-related fatalities, particularly in endemic regions [3]. This pervasive challenge significantly hampers the accurate measurement of malaria-specific mortality rates at the population level.

To address these challenges, verbal autopsy (VA) methodology has been developed to ascertain the cause of death (CoD) at the population level. VA involves administering a structured questionnaire to a family member or someone closely associated with the deceased, collecting information on signs, symptoms, and other events leading to death [3]. According to the World Health Organization (WHO) Verbal Autopsy Reference Group [4], the WHO has been continuously developing improved VA instruments since the 1970s though it originated from Africa and Asia in the late 1950s. In the early 1990s, the WHO developed standards on how to conduct a verbal autopsy, developed three more VA models in 2007, and then updated and refined in 2012, 2014, 2016, and 2022 [5]. VA methodology is universally acceptable and can be conducted closer to the moment of death, weeks or even months later.

Despite its widespread use, VA methodology has inherent limitations. These include reliance on potentially incomplete or biased information from relatives and caregivers, subjective interpretation by interviewers and physicians, the need for standardized training, and limited diagnostic accuracy [6]. Also, VA cannot differentiate between the presence of a given pathogen (infection) and the pathogen actively causing disease and contributing to death (pathogenicity). These limitations can lead to significant challenges in accurately identifying the true cause of death. Complete Diagnostic Autopsy (CDA) stands as the gold standard due to its ability to provide definitive evidence on CoD, contingent upon the availability of resources, infrastructure, and cultural acceptance by the general population [7]. However, CDAs are quite uncommon. VA lacks reliance on clinical and laboratory findings, potentially leading to misclassification errors. As elucidated by Anker [8], the impact of misclassification on observed estimates of mortality attributed to specific causes, such as malaria, is influenced by two primary factors: (i) the sensitivity and specificity of the VA instrument; (ii) the true proportion of deaths from that cause (the cause-specific mortality fraction).

There has been limited investigation into the validity of VA for malaria deaths. One of the predominant constraints in utilizing VA for assessing malaria-related mortality, pertains to its low levels of sensitivity and specificity [3, 8]. Furthermore, there exists a potential for VA data to either underestimate or overestimate the true cause-specific mortality fraction attributed to malaria [3, 8].

In an effort to address this evidence gap, the Child Health, and Mortality Prevention Surveillance (CHAMPS) network has been conducting standardized mortality surveillance in seven countries of which six are in Africa [9]. CHAMPS endeavours to discern definitive causes of death and curb child mortality rates through the implementation of Minimally Invasive Tissue Sampling (MITS) autopsies on eligible deaths of children under the age of five. MITS is a detailed post-mortem procedure involving needle-based sampling of highly informative organs in the body by trained pathology technicians for detailed microbiological and pathological analysis [10]. It has been acclaimed as an acceptable alternative to the gold standard CDA and is more acceptable in LMICs.

Other studies that applied the VA methodology have produced U-shaped malaria mortality-by- age curves with high mortality rates among young children, low rates among adolescents and young adults, and higher rates in adults 45 years and older [7, 11, 12].

In malaria-endemic countries, older children, adolescents, and adults gradually develop partial naturally acquired immunity (NAI) against malarial disease due to the repeated exposure to malaria parasites over time from the infective bites of female *Anopheles* mosquitoes yielding sufficient strain-specific immune responses [13]. Since younger children are still developing NAI, they remain highly susceptible to clinical malaria and its adverse outcomes.

The purpose of this systematic review is to provide a concise synthesis of relevant literature, focusing on the validation of malaria-related deaths, particularly among children under the age of five, by comparing VA diagnoses with those obtained through MITS and/or CDA methodologies.

## Methods

This systematic review focused on original studies which had assessed and reported on the validity of VA compared to MITS and / or CDA, and which provided evidence applicable to deaths from malaria, mainly in children. This study was registered with the International Prospective Register of Systematic Reviews (PROSPERO #: CRD42023452940) [14].

All studies that presented validity assessment for VA in relation to MITS and / or CDA were identified. Diagnostic test accuracy studies with a validation component were included whilst non-validation studies were excluded. There were no restrictions by type of setting whether clinical or community.

A comprehensive literature search for original studies was conducted in Ovid MEDLINE, Ovid Embase, CINAHL via EBSCO, Scopus, and The Cochrane Library via Wiley from 1st January 1990 to 31st March 2024. Input and guidance were obtained from a librarian at the University of Toronto in finalizing the search. The strategy was designed to maximize recall of relevant articles over specificity. Keywords were searched in article titles, abstracts, and body (where available in database) in addition to indexed terms to include sources without indexing. Citation mining was done to identify similar papers, both backward and forward iterative approaches were conducted. Also, the authors of relevant publications were used in author-based searches to identify additional relevant studies. The review focused on articles published only in English Language.

The search terms or keywords included three different concepts across three layers. The search terms were “malaria” or “*plasmodium falciparum*” or “paludism” and “verbal autopsy” or “post-mortem interview” or “mortality surveillance” and “Minimally Invasive Tissue Sampling” or “Minimally Invasive Autopsy” or “Complete Diagnostic Autopsy”. Subject heading searches (e.g., MeSH, Emtree terms) were included in the strategy. To expand the search and maximize the likelihood of finding relevant validation and measurement-focused studies, other available network websites especially the MITS surveillance alliance and Google Scholar were searched to identify additional programme reports, articles, and grey literature on malaria-specific mortality, even though published studies are the priority.

The study selection consisted of two rounds. First, databases and websites were searched, and all titles and abstracts of records were screened by two of the authors (RC-M & PJ) to determine if they met the inclusion criteria, i.e. whether they are related to predictions of individual causes of death or malaria cause-specific mortality fractions by VA, MITS and CDA; those deemed not relevant were verified for exclusion. Second, full texts of publications that passed the screening were reviewed to determine eligibility i.e., whether any validation component was present. The study methodology has been reported using the Preferred Reporting Items for Systematic Reviews and Meta-Analyses (PRISMA) guidelines.

The quality and applicability of each included study was assessed and evaluated using the Quality Appraisal of Reliability Studies (QAREL) checklist [15]. The checklist has 11 items that explore seven principles. The assessment was summarized using a colour code system.

There were 16 variables extracted from the selected studies. They are: (1) setting/location of the study; (2) VA instrument used in the study; (3) place of interview; (4) time between death and VA interview; (5) time between death and MITS / MIA; (6) time between death and CDA; (7) description of the deceased; (8) age composition; (9) gender composition; (10) sample size/no. of death records collected; (11) source of details of the deceased; (12) whether comparator was a primary or secondary data source; (13) methods used for determining COD in the study; (14) criteria used to determine a malaria death in the study; (15) measures used in describing concordance; (16) findings of the concordance analysis.

A narrative synthesis of the publications that met the inclusion criteria was done. The reviewers carried out a thematic comparative analysis of measuring malaria and other mortality through VA and other comparators mainly MITS and CDA.

## Results

### Inclusion strategies

**Fig. 1:**
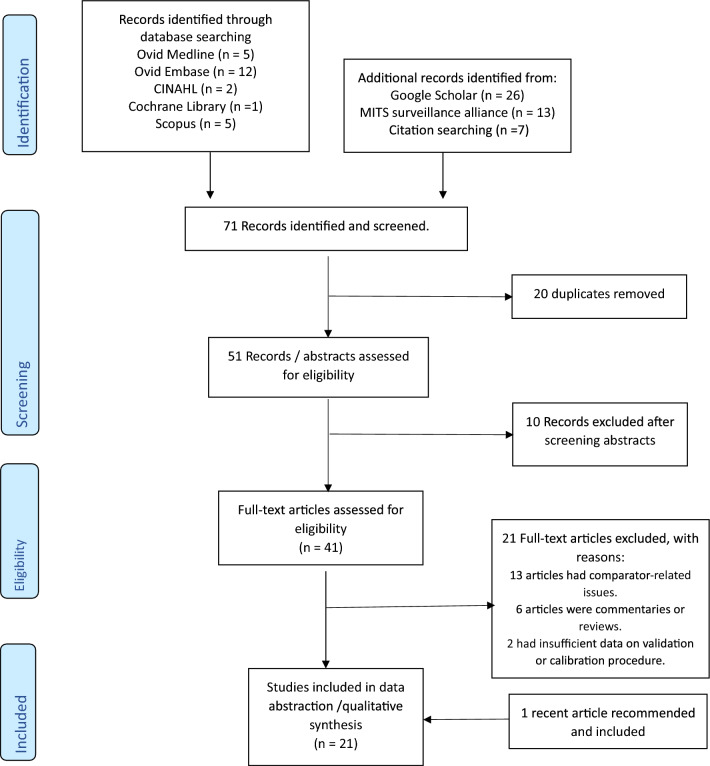
Flow diagram of publication screening and identification

A total of 71 papers were first identified in which 25 were identified through database search using the three different concept combinations, and 39 were identified through the MITS surveillance alliance site and Google scholar. Seven additional studies were included through citation mining. There were 20 duplicates leaving 51 records eligible. After reading the abstracts, ten were excluded, 41 full text articles were then assessed. One recently published article that met the eligibility criteria was recommended and included. A total of 21 articles were finally included in the review. See Fig. 1.

The main characteristics of the identified publications were extracted (additional file 1) and the list of the 16 variables has been mentioned above in the methods section.

### Summary of articles

There were five articles that had full VA validation studies [16–20]. Only one [20] had physician coded verbal autopsy (PCVA), the rest were computer coded verbal autopsies (CCVA). Since MITS process involves a VA component that contributes to its final COD, its studies were included since VA validation studies in relation to MITS are so few. There were eight MITS validation studies [21–28] and seven MITS focused studies [9, 13, 29–33]. One of the VA validation studies [16] also has a MITS validation component and one of the MITS focused studies [13] had a VA validation component. One article focused only on CDA [34]. Ten of the studies dealt purely with hospital facility deaths [16, 17, 21–24, 26–28, 34] whilst the other 11 had combined community and facility deaths [9, 13, 18–20, 25, 29–33].

#### Location of the study

Most of the studies included in this review have been conducted in various CHAMPS sites within seven countries. These are Bangladesh, Ethiopia, Kenya, Mali, Mozambique, Sierra Leone, and South Africa. Six of these included publications are MITS focused studies that came from all seven CHAMPS sites [9, 13, 29, 31–33]. There were ten studies that were conducted only in Maputo Central Hospital, Mozambique [16–19, 22–24, 26–28], this brings the total number of studies from Mozambique to 16. There were two studies from Brazil [20, 21], one separately from Ethiopia [30] thus bringing Ethiopia’s contribution to seven, one from Astana, Kazakhstan [25], and one from Mumbai, India [34].

#### Study populations and timing of the procedure

All death categories from still births to adults were represented in the various study populations, however most studied neonates and children since CHAMPS work focuses more on under-five year old children. Five articles included maternal deaths in their study sample and four other articles included non-pregnant women of child-bearing age. The total number of deaths studied across the 21 articles is 19,388. The VA instrument most used in the studies were either 2012 or 2016 WHO VA questionnaire. VA data were mostly collected within four weeks after death. The MITS samples were mostly collected within 24 h of death or up to 36 h if the body was preserved through refrigeration. The CDA procedure was mostly done within 24 h after the MITS procedure or clinical data extraction.

#### Analysis techniques

The measures mostly used to compare the methods included descriptive statistics, sensitivity, specificity, positive and negative predictive value, and Kappa statistic. Almost all the studies used multiple measures. Sensitivity, specificity, positive and negative predictive values were used in eleven studies [13, 16–24, 26], Kappa statistic was used in seven studies [17, 21, 23–27], and chance corrected concordance (CCC) was used in two studies [17, 20]. Cause-specific mortality fraction (CSMF) was used in four studies [13, 17–19], CSMF accuracy (CSMFA) and chance-corrected CSMFA (CCCSMFA) were used in two studies [17, 20]. Eleven studies used mean, median, frequencies and percentages [9, 21, 23, 25, 28–34], χ2 analysis was done in five studies [9, 29, 32–34], McNemar’s test was done in two studies [25, 27], Fisher’s exact test and odds ratios (ORs) were used in three studies [22, 32, 33].

#### Quality of the study

The quality and applicability of the included diagnostic accuracy studies were assessed using the QAREL checklist (additional file 2). The overall quality of all the included studies was good. None of the studies recorded a ‘no’ response which would have caused the study to have a poor quality. For items 5 – 7, the answer ‘not applicable’ was applied for those MITS focused studies since the clinical data and VA information were required to arrive at the COD and there was no reference standard in these studies.

### Malaria mortality VA validation compared with MITS

Three studies were fully focused on malaria [13, 16, 34]. Malaria was mentioned as part of the diagnoses and thus part of the validation in eight studies [8, 17–19, 22–24, 29].

Malaria was screened for in almost all the studies. The various laboratory tests used for the screening and detection of the malaria parasite included rapid diagnostic test, blood smear microscopy and using real-time quantitative polymerase chain reaction (qPCR) assay. CHAMPS sites additionally do molecular analysis conducted through TaqMan Array cards (this is a customizable diagnostic platform that allows the performance of dozens of real-time PCR reactions simultaneously (TAC); ThermoFisher Scientific, Waltham, MA, USA) [13]. In two studies [16, 17], further search for *P. falciparum* was conducted through histologic examinations, immunohistochemical stains and also thorough search for haemozoin in macrophages was conducted in all tissues as evidence of the past malaria infection. Malaria was assigned as a COD based on: (a) presence of cerebral malaria or (b) presence of abundant haemozoin deposition in tissues in the absence of other CoD [16, 17]. In the CDA focused study done in India [34], malaria was diagnosed if the ring, schizonts and/or gametocytes of the malaria parasite were identified either before death or in the post-mortem peripheral blood smears or by the presence of malaria pigment in the splenic imprint or in the tissues collected for histopathological examination. It is worthy to note that very high parasitaemia adds credibility to the importance of malaria in the causal pathway to death while very low parasitaemia are less indicative of causality.

In a CHAMPS study [9], malaria accounted for 16% (39 out of 241) of child (age 1 to 5) deaths when only the underlying cause was considered and 5% (13 out of 278) of infant deaths. However, when the full causal chain was considered, malaria accounted for 27% (66 out of 238) of child deaths indicating a 1.7-fold increase.

In another CHAMPS study [29], malaria was one of the six most common underlying COD at 11% (71 out of 632 decedents) in children 1 to 59 months. When the full chain of events was considered, malaria accounted for 20% (123 decedents) of child deaths. It is important to note that not all the seven CHAMPS sites had malaria deaths, it was absent from South Africa, Ethiopia, and Bangladesh sites.

Malaria was responsible for 21% of the febrile deaths in a CDA focused study done in Mumbai, India [34]. Malaria deaths mostly occurred during the monsoon period or rainy season. *Plasmodium falciparum* was mostly responsible for these deaths and the most common complication or mode of death was cerebral malaria in this study [34].

In a recent article by Ogbuanu et al*.* [13], malaria accounted for 30.5% (262/858) of the deaths in children aged 1 – 59 months in four countries (Sierra Leone, Kenya, Mozambique and Mali). Sierra Leone had the highest malaria-associated deaths with 42.9% (126/294), followed by Kenya with 31.4% (96/306), Mozambique with 18.2% (36/198) and Mali with 6.7% (4/60) malaria-associated deaths in the full causal chain [13]. No malaria-attributable death was documented among stillbirths and neonates. All these malaria-associated deaths were attributed to *P. falciparum* however, there was also a high bacterial co-infection (24%). The most likely comorbid condition seen in these malaria cases was anaemia.

**Fig. 2:**
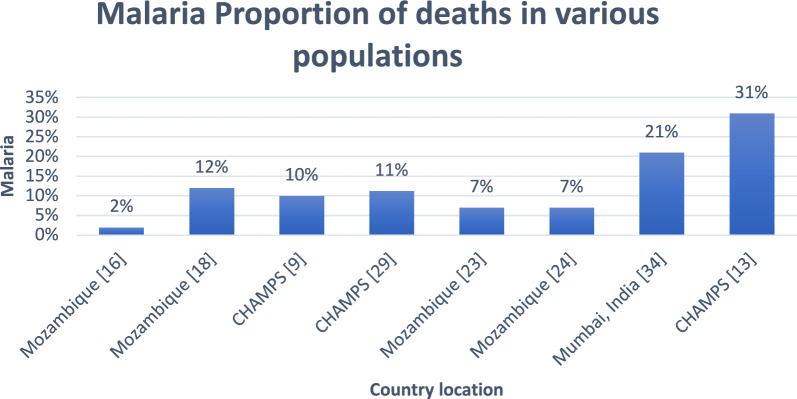
Malaria proportion of deaths in various country locations

See Fig. 2 for the malaria proportion of deaths in various country locations. In one of the studies conducted in Mozambique [16], out of 264 deaths, only 6 (2%) were due to malaria. Using the CDA procedure as the gold standard, the sensitivity and specificity of the VA were 33% and 96% and for the MITS, it was 100% and 100%, respectively. Another study [17] also found a low sensitivity of 33% and a specificity of 97% in identifying malaria using VA (InterVA model) in comparison to the gold standard CDA.

The recent CHAMPS study [13] with a correlation component between computer-coded VA and MITS results for malaria diagnosis also found low levels of sensitivity and moderate to high levels of specificity. In the detection of malaria as a CoD based on MITS, VA analysis using the InSilico method had a sensitivity and specificity of 29% and 86.6%, respectively, whereas the InterVA method had a sensitivity and specificity of 18.4% and 95.1%, respectively [13]. The sensitivity and specificity of the VA validation studies above ranged from 18.4% to 33% and from 86.6% to 97%, respectively.

In two multi-cause calibration studies [18, 19], both InSilicoVA and Expert Algorithm VA (EAVA) exhibited high frequency of misclassification for malaria in comparison to MITS even though malaria was identified more often in InSilico VA than EAVA. For InSilico VA, malaria was correctly identified in 48% of the multi-cause MITS malaria deaths. The MITS—coded malaria deaths were misclassified by VA as other infections and pneumonia in 24% and 21% of the deaths respectively [18]. For the EAVA, malaria was correctly identified in only 12% of the multi-cause MITS malaria deaths. The MITS—coded malaria deaths were highly misclassified by VA as other infections and pneumonia in 35% and 34% of the deaths, respectively [18]. These results are similar to the study also conducted by Fiksel et al*.* [19]. When the data were calibrated, the malaria CSMF estimates for children (1 – 59 months) for the three VA methods including ensemble model increased [18, 19].

### MITS validation compared with CDA

MITS was validated against CDA in nine studies across all age groups. The MITS procedure has been shown to have a high sampling success rate ranging from 67% for the kidney to 100% for blood, CSF, lung, liver, and brain [28]. These organs have the most diagnostic yield. In a MITS validation conducted by Castillo et al*.* in adults [22], there was 76% (85 / 112) overall concordance between the MIA diagnosis and CDA diagnosis. Infectious diseases (79%) and malignant tumours (81%) had higher concordances than other diseases (56%). In the MITS samples, there was also a higher identification rate (84%) of specific microorganisms causing deaths due to infectious diseases. In another MITS validation conducted by Bassat et al. [24] in children, a substantial concordance was seen with the CDA (Kappa = 0.70, 95% CI 0.49–0.92) and the agreement was 75% (36 / 48) of the cases. As with other studies, the MIA or MITS showed a high sensitivity and specificity for infectious diseases (93% and 75%) and malignant tumours (100% and 100%, respectively.

A study in Manaus, Brazil [21], yielded a degree of coincidence or overall concordance of 85% (47 / 55 cases) between the MIA and the CDA diagnoses (Kappa = 0.78, 95% CI 0.68–0.95). This perfect level of coincidence was similarly higher in infectious diseases (90%) and malignant tumours (87%) than for other diseases (67%). There was also a high concordance of 83% when MITS diagnosis in neonates and infants were compared with CDA diagnosis based on the full causal chain in Nur-Sultan, Kazakhstan [25].

In summarizing the studies contributing to this theme, overall concordance rates between MITS diagnosis and CDA diagnosis ranged from 68% in maternal deaths [23] and in neonates [26] to 85% in predominantly adults [21] and 90% concordance in disease categorization. MITS was shown to be relatively accurate and reliable, thus a robust substitute for the CDA.

### Importance of clinical information

In all the MITS validation studies listed above, the results were analyzed blindly to clinical data to know how accurate the technique was. In order to know how valuable the clinical data to the diagnostic yield of the MITS is, Fernandes et al*.* [27] analysed MITS blinded to clinical data (MITSb), MITS enhanced with clinical data (MITSc), CDA blinded to clinical data (CDAb) in comparison to the gold standard CDA with clinical data (CDAc). They found out that clinical data increased diagnostic coincidence between MITS blind to clinical data and the gold standard by 11% (30 / 264 cases) and modified the CDAb diagnosis in 20 (8%) of 264 cases. There was a significant increase in concordance between MITSb and MITSc with the gold standard in neonates, adults, and maternal deaths. In children, even though the increase in k value was not significant, it was still evident. The agreement between MITSb and CDA for children was 89% (48/54 cases; κ value = 0.704, substantial agreement) [27]. When the clinical data were added (MITSc) in comparison with CDA, the degree of coincidence increased to 93% (50/54 cases; κ value = 0.802, almost perfect agreement). Overall, the addition of clinical data increased the diagnostic accuracy of MITS in relation to CDA.

## Discussion

Despite a rigorous search, this systematic review found limited recently published articles on the validation of VA in comparison to MITS and CDA. The malaria CSMFs across various studies ranged from 2 to 31%. This wide range of malaria CSMFs is due to multiple factors such as variability in malaria epidemiology, environmental factors, population demographics, seasonal variations, health system differences, bias and diagnostic criteria. In the first round of a nationally representative mortality survey conducted in Sierra Leone using PCVA, malaria was the leading cause of death in children and older adults thus accounting for 22% [7]. A study conducted in Western Kenya by Amek et al*.* [35] revealed similar PCVA findings. The VA COD attributable to malaria was 21% in under five-year-old children albeit with low sensitivity (26%) and moderate specificity (85%) [35]. The latest CHAMPS study [13] also found that Sierra Leone has the most malaria-associated deaths in all the CHAMPS sites followed by Kenya. CHAMPS is currently conducting a study in Bo, Sierra Leone on adult malaria mortality which will, for the first time, and hopefully unequivocally, shed some light on the claims (the “U-shape” malaria mortality-by-age curve debate) that malaria may be an important cause of adult mortality in malaria-endemic settings.

The levels of sensitivity of the VA versus MITS / CDA regarding malaria-attributed deaths were as low as one-third or less and the levels of specificity were as high as nine-tenths. There was a high level of misclassification for both InSilico and EAVA for malaria compared to MITS [18, 19]. The studies in a systematic review by Herrera et al*.* [3] also found a varying range of low levels of sensitivity and moderate to high levels of specificity. The observed disparities in accuracy may be attributed to several underlying factors. Fever has been a cardinal sign of malaria and historically, it has been synonymous with malaria in malaria-endemic countries. However, the signs and symptoms of malaria are not pathognomonic; hence, other infectious diseases or febrile illnesses, such as pneumonia, septicaemia, meningitis, can be misclassified as malaria. Misclassification bias can also be influenced by the malaria epidemiological context and the experience and knowledge of the physicians coding of mortality records [3]. Malaria mortality can be overestimated in high transmission areas wherein all acute febrile illnesses are suspected to be malaria and it can also be underestimated in low transmission areas. Previous VA validation studies were compared with hospital records. These diagnostic studies with hospital records comparator were not included in this systematic review. In malaria endemic areas, such ‘gold standard’ comparator may misclassify the cause of death since asymptomatic parasitaemia is common [36]. This comparator may also pose data quality challenges and have a different reference population from the actual study population [3]. Diagnostic pathology autopsies are the true gold standards for the determination of COD.

The accuracy of a VA estimate of the proportion of deaths attributed to malaria is inversely proportional to the difference between the true population of malaria deaths and the VA estimate. The smaller the difference between the true population of deaths from malaria and the VA estimate of the proportion of deaths from malaria, the more accurate the estimate and the larger the difference is, the less accurate the estimate [8]. It is highly important to note that the accuracy of a VA estimate is usually much more dependent on the specificity of the VA instrument rather than the sensitivity [8]. Considering the low sensitivity and high specificity regarding malaria-attributed deaths, one may hypothetically look at different scenarios of sensitivity and specificity and its impact on the CSMF using the equation proposed by Anker [8].

The VA is a correct estimate if:

$${\text{a }} + {\text{ b}} = {1 }{-}{\text{ specificity}}$$

$${\text{N }} {2}{-}{\text{specificity}}{-}{\text{sensitivity}}.$$

The VA is an underestimate if a + b /N is greater than the equation on the right-hand side and an overestimate if a + b/N is lesser.

It becomes apparent that when the sensitivity is doubled while keeping specificity constant, a substantial overestimate of the CSMF results. Similarly, a significant overestimate also occurs when the sensitivity remains constant, and the specificity is decreased modestly by 2%. Doubling sensitivity has an impact equivalent to a modest reduction in specificity. Therefore, maintaining high specificity is more important. This shows that results of VA studies with high specificity in malaria endemic areas are largely plausible.

MITS performed better when compared to CDA in ascertaining COD based on the overall concordance rates, with the highest concordance seen in deaths due to infectious diseases and malignant tumours. This finding fully coincides with the results of the narrative review by Paganelli et al*.* [10]. Also, MITS has been shown to have higher sensitivity and specificity in identifying malaria-specific mortality when compared to CDA. Since MITS is a feasible and reliable method for postmortem examination when compared with CDA, it is recommended that MITS method is expanded to other LMICs, and more funders are available to support its implementation.

MITS has generally shown better accuracy than VA in diagnosing malaria attributable deaths. However, VA is more useful to reach those deaths occurring at home though its performance may be still lower in comparison to MITS or CDA. Most of the deaths assessed in the studies in this review are hospital deaths (hospitalized patients) which are different from community deaths (community populations) especially when the populations differ by socioeconomic level. According to the Healthy Sierra Leone (HEAL SL) report (2018—2023) [37], 52% of the deaths occurred at home and 39% died in hospital / health facility. MITS has several limitations when it comes to assessing malaria-attributable deaths that occur at home. The procedure should be done in a well-controlled setting since the home environment may pose logistical challenges and resource limitations. The timely execution of MITS procedures may be compromised when deaths occur at home, as there may be delays in notifying healthcare authorities and arranging for the procedure, potentially affecting the quality of tissue samples and diagnostic accuracy. The quality of tissue samples obtained through MITS at home settings may be variable due to factors such as post-mortem changes, environmental conditions, and the expertise of personnel conducting the procedure, which can impact the reliability of malaria diagnosis [9, 28]. MITS is not practicable at scale and has biases in enrolled populations. Also, obtaining comprehensive information about the deceased's medical history and symptoms preceding death may be more challenging for home deaths, affecting the interpretation of MITS results. Therefore, MITS and CDA may be limited in their diagnostic accuracy for home deaths.

This review also documented that the addition of clinical data increased the diagnostic accuracy of MITS by one-tenth (11%) in comparison with the gold standard CDA. Both the MITS with or without clinical information and the CDA blind to clinical information had good correlation to the gold standard which resulted in a significant increase in concordance. VA data also provides a good amount of information collected from the respondent which guides physician coders, computer algorithms and recently Artificial Intelligence (AI)/ large language models to arrive at a suitable underlying COD. The HEAL SL surveillance program now uses OpenAI ChatGPT—4 to support physician coding of VA records in addition to InSilico VA and Inter VA [37]. VA information from facility or community deaths with clinical and laboratory information provides a better guide than medically unattended deaths or community deaths with no clinical information since respondents may have learnt about the cause of death from the hospital.

A strength of this systematic review is that the VA studies were compared to the true gold standard CDA whilst previous reviews included studies that used hospital records as the comparator. The use of hospital records or clinical diagnosis has its challenges. Second, the study populations in the different studies included both deaths from hospitalized patients and from the community. Third, the VA tools used in the studies were mostly standardized since the WHO 2012 or 2016 VA standard format was used. Fourth, the PRISMA guidelines were followed to ensure methodological standards [38].

There are limitations of this review. First, the COD determination for both the VA methodology and MITS required some degree of subjective interpretation though it is higher for VA. Second, some of the validation studies have a relatively small sample size which leads to sensitivity and specificity estimates with low precision. Third, even though all age groups were included in the studies of this review, neonates were excluded from the malaria COD analyses since malaria is unlikely to be the underlying COD in this age group. This is mainly due to the protective effect of maternal immunoglobulin G (IgG) malaria-specific antibodies acquired by the fetus via the placenta in utero though it decreases during the first year of life [39]. Studies with neonates would differ from those without neonates regarding the sensitivity, specificity, and proportion of deaths attributed to malaria by verbal autopsy. Fourth, only articles in English were included in the review. Fifth, 17 out of 21 studies included in this review originated from the same countries, which may limit generalizability.

## Conclusions

This systematic review revealed a paucity of literature on the validation of malaria-attributed deaths using VA in comparison to MITS and CDA. The malaria CSMFs in the various studies widely ranged from 2 to 31%, reflecting the diverse epidemiological and geographical contexts, age groups and diagnostic criteria for malaria deaths across different studies. *Plasmodium falciparum* was mostly responsible for these deaths and the most common complications or modes of death were anaemia and cerebral malaria. VA studies have revealed varying levels of low sensitivity and moderate to high specificity, influenced by factors such as the malaria epidemiological context, physician coding experience, and the inherent challenges of attributing malaria as the cause of death.

The overall concordance rates between MITS and CDA diagnoses ranged from 68 to 90%, with the highest concordance seen in deaths due to infectious diseases and malignant tumours. MITS demonstrated better accuracy compared to VA in diagnosing malaria-attributed deaths, particularly in hospital settings. Furthermore, the addition of clinical data has been shown to enhance the diagnostic accuracy of MITS by 11%, emphasizing the importance of comprehensive information in guiding COD determination. The high specificity of malaria in VA diagnosis suggests population-based estimates of the proportion of deaths due to malaria are broadly plausible. Hence efforts to improve VA by using MITS calibration are justified from a public health perspective.

This robust review suggests that further VA studies in malaria endemic areas with MITS and CDA studies in selected populations are highly required to inform policy and practice. By enhancing the overall understanding of malaria mortality, advancements can be made in the measurement of malaria-related deaths, thereby contributing to more effective public health interventions aimed at reducing global malaria mortality.

### Supplementary Information

Supplementary Material 1. Data extraction excel sheet and Completed QAREL checklist.

Supplementary Material 2. List of Included and Excluded articles

## Data Availability

All data relating to the present study are available in this manuscript and Additional files.

## Abbreviations

CCC: Chance corrected concordance
CDA: Complete Diagnostic Autopsy
CHAMPS: Child Health, and Mortality Prevention Surveillance
CoD: Cause of death
CSMF: Cause specific mortality fraction
LMIC: Low- and middle-income countries
MITS: Minimally invasive tissue sampling
NAI: Naturally acquired immunity
PCR: Polymerase chain reaction
PRISMA: Preferred Reporting Items for Systematic Reviews and Meta-Analyses
QAREL: Quality Appraisal of Reliability Studies
VA: Verbal autopsy
WHO: World Health Organisation
